# Changing Patient Profile in a Psychiatric Hospital During COVID Pandemic: A Comparison With Pre-COVID State

**DOI:** 10.1192/bjo.2022.395

**Published:** 2022-06-20

**Authors:** Nilamadhab Kar, Stephen Amarjeet Jiwanmall

**Affiliations:** Black Country Healthcare NHS Foundation Trust, Wolverhampton, United Kingdom

## Abstract

**Aims:**

COVID-19 pandemic has a massively adverse mental health impact and people with pre-existing psychiatric illnesses are one of the most severely affected groups. We intended to study the changes in the patient profile in a psychiatric hospital during the COVID-19 pandemic, comparing it to the period just before the pandemic.

**Methods:**

Consecutive patients (n = 210) admitted to psychiatric ward under one team during COVID-19 pandemic (February 2020 to January 2022) were compared with patients (n = 234) admitted in the immediate pre-pandemic period (January 2017 to January 2020). Demographic (age, gender, and ethnicity) and clinical variables (diagnosis, admission days, Mental Health Act status, risk to self and others) were collected from the electronic patient records and analysed.

**Results:**

During the pandemic monthly admission rates have gone up by 38.1% over the base rate of 6.32/month. There was no difference in the mean age at admission; or the proportion of patients aged 18–40 years or above in the pre-pandemic and pandemic groups. Similarly the gender composition of patients in the two periods was comparable. Proportion of patients from Asian background increased from 7.7% to 16.8% during pandemic period (p < 0.05). The number of hospital days decreased from 31.97 ± 45.8 days in the pre-pandemic period to 22.44 ± 25.1 days during pandemic (p < 0.05). Along with increased admission rates, it suggested a rapid flow of the admission and discharge during the pandemic. Considering diagnostic composition between pre-COVID-19 and COVID-19 periods, psychotic (27.8% v 26.7%) and mood disorders (18.8% v 23.3%) were the predominant; and substance related disorders (20.5% v 16.7%) were the most common comorbidities. Risk to self was associated with 84.3% admissions during the pandemic compared to 78.6% in the pre-pandemic period; however, risk to others was noted in 13.8% v 22.2% (p < 0.01) respectively. There was no difference in proportions getting admitted under Mental Health Act or being discharged with Community Treatment Order. Interestingly, proportions of patients getting discharged under the care of Home Treatment Team decreased from 31.1% pre-pandemic to 16.5% during pandemic period (p < 0.005).

**Conclusion:**

There is an increase in admission rate and decrease in the number of admission days, suggestive of increased demand of clinical resources during pandemic. This could be reflective of the stressful situation and adverse impact on mental health in the pandemic period. As the impact on mental health is expected to continue, there is a need for greater resources both in community and inpatient psychiatric services.

